# Crystal Structure of Photosystem I Monomer From *Synechocystis* PCC 6803

**DOI:** 10.3389/fpls.2018.01865

**Published:** 2019-01-04

**Authors:** Sigal Yoli Netzer-El, Ido Caspy, Nathan Nelson

**Affiliations:** Department of Biochemistry and Molecular Biology, The George S. Wise Faculty of Life Sciences, Tel Aviv University, Tel Aviv, Israel

**Keywords:** cyanobacteria, membrane proteins, photosystem I (PSI), electron transport (photosynthetic), photosynthesis

## Abstract

A single histidine addition to the C-terminus of PsaL of *Synechocystis* sp. PCC 6803 was previously reported by our lab to shift the trimer-to-monomer ratio of PSI in favor of the monomeric form. P700 re-reduction and NADP^+^ photo-reduction measurements of the PsaL^HIS^ strain show no effect on PSI activity in comparison to the WT trimeric PSI. Crystal structure of the PsaL^HIS^ monomeric PSI reveals several alterations that occurred in the trimerisation site of PSI, primarily a deformation of the C-terminus of PsaL and loss of chlorophyll a and β-carotene molecules.

## Introduction

Oxygenic photosynthesis provides the necessary food and fuel for sustaining life on earth, by converting the sunlight energy into chemical energy available for all living creatures (Barber, 2004; Nelson, 2011). This intricate process takes place in photosynthetic organisms and is divided into a light dependent stage and a light independent stage. In the light dependent stage two large, multi-subunit, pigment-protein complexes known as Photosystem I and II (PSI and PSII), are embedded in the thylakoid membranes, absorb light and turn physical energy into energy stored in chemical bonds. PSII mediates light-dependent water oxidation, which supplies electrons and protons to the photosynthetic process and releases oxygen, while PSI mediates NADP^+^ photo-reduction (Nelson and Yocum, 2006).

A major portion of the world’s photosynthesis occurs in the ocean and is performed by single celled mesophilic cyanobacteria and algae. The structure of both PSI and PSII was determined in thermophilic cyanobacteria at high resolution, 2.5 and 1.9 Å, respectively (Jordan et al., 2001; Umena et al., 2011). Recently our group published a high resolution structure of trimeric PSI at 2.5 Å from the mesophilic cyanobacterium *Synechocystis* sp. PCC 6803 using x-ray crystallography (Malavath et al., 2018). This model shows the architecture of trimeric PSI (nearly 1 mega-Dalton) and consists of 33 protein subunits, 72 carotenoids, 285 chlorophyll a molecules, 51 lipids, 9 iron-sulfur clusters, 6 phylloquinones, and 6 putative calcium ions.

In cyanobacteria, PSI is found in a trimeric or monomeric form, though recently has been reported to appear also in a tetrameric form (Watanabe et al., 2011, 2014; Li et al., 2014; Semchonok et al., 2016). Under optimal growth conditions, the trimeric form of *Synechocystis* PSI is more abundant, whereas the monomeric form is present as a smaller fraction of the total complex. However, under different growth conditions, such as light, temperature and available nutrients the ratio of monomers to trimers may shift toward the monomeric form (Ivanov et al., 2006; Salomon and Keren, 2011; Kłodawska et al., 2014). The preference of one form over the other depending on environmental conditions suggests that each form functions slightly different, which may translate into changes in structure. Moreover, in our recently published PSI trimer model we showed a deviation from symmetry in which each monomer within the trimer holds a different set of ligands. It is still unclear whether the trimer is built from a homogenous pool of monomers that are modified later on to their specific ligand set, or three different monomer types are generated and then fused together to form the asymmetric trimer (Malavath et al., 2018).

To investigate the *Synechocystis* PSI monomer we performed a single histidine addition to the C-terminus of PsaL to generate a PsaL^HIS^ mutant strain (Malavath et al., 2018). The mutated strain generated mostly monomers (monomer-to-trimer ratio is 4.5:1), which allowed us to examine the monomer structure more easily, now constituting the major fraction of PSI, while taking specific interest in the trimerisation site. The crystals generated from PSI of PsaL^HIS^ were solved to 4 Å resolution and showed some differences in PSI structure, in comparison to WT PSI, primarily in the region of PsaL, and PsaI. Here we will present the structure of monomeric PSI and discuss the differences between the WT and the mutated PsaL^HIS^ models.

## Materials and Methods

### PsaL^HIS^ Thylakoids Preparation and Monomer PSI Purification

The PsaL^HIS^ construction, thylakoid preparation and PSI purification were described previously in Malavath et al. (2018). Briefly, PsaL^HIS^ strain membranes were thawed and incubated with n-Dodecyl β-maltoside. After centrifugation, the sup was loaded onto a Toyopearl DEAE 650-C column. Green fractions were collected and precipitated with 100 mM NaCl and 10% PEG 3350. The re-suspension was loaded on a sucrose density gradient, dark green bands collected, and loaded on a SOURCE^TM^ 15Q column. Dark green fractions were collected, loaded onto a sucrose density gradient and ultra-centrifuged. The dark green bands were collected from the gradients and used for crystallization. PSI trimer from the WT *Synechocystis* sp. PCC 6803 was purified by the standard procedure described in Malavath et al. (2018), except that only one column and one sucrose gradient were used during the purification. Briefly, WT strain membranes where thawed, and incubated with n-Dodecyl β-maltoside. After centrifugation, the sup was loaded onto a Toyopearl DEAE 650-C column. Green fractions collected and precipitated with 100 mM NaCl and 10% PEG3350. The re-suspension was loaded on a sucrose gradient, and dark green bands collected.

### PSI Crystallization and Cryogenic Protection

Crystallization was carried out by the Sitting drop variant of the vapor-diffusion technique at 4°C (Charles Super Company). Aliquots (4 μl) of PSI solution were mixed with equal volumes of a reservoir solution containing 160 mM KCl, 40 mM MES-NaOH (pH 6.5), and PEG 3350 (final content, 8–10%). One of two additives was used to improve the resolution: either 0.01% sulfoquinovosyldiacylglycerol (SQDG, Avanti) or 5% Jeffamine M-600 pH 7 (Hampton Research). Dark green crystals appeared after 3 days at the higher PEG concentrations, but the best diffracting crystals appeared after 2–3 weeks at the lower PEG concentrations. For cryogenic protection the crystals were soaked in a solution of 160 mM KCl, 40 mM MES-NaOH (pH 6.5), and increasing PEG 3350 concentrations of 10, 15, 20, and 30%. Crystals were sent for MS analysis to verify the presence of PsaL^HIS^, as described in Malavath et al. (2018).

### Data Collection and Processing

The crystals were immediately frozen in liquid nitrogen. X-ray diffraction data were collected at the European Synchrotron Radiation Facility (ID23-2), the Swiss Light Source (PXI), and BESSYII (14.1). The data were analyzed, processed, and refined, as previously described by Mazor et al. (2014). Five data sets were combined using the AIMLESS suite for CCP4 to generate a 4 Å dataset (Winn et al., 2011). STARANISO server was used for Anisotropicity corrections (Tickle et al., 2016).

### PSI Kinetic Measurements

Plastocyanin of *Synechocystis* sp. PCC 6803 was heterologously expressed as described by Hulsker et al. (2007). The codon-optimized petE gene was delivered by IDT and ligated into expression vector pET17b (Invitrogen). The 28-amino acid transit peptide of petE was deleted and the following amino acid was replaced by a single methionine. Ferredoxin and FNR were produced and purified from Swiss chard (Nelson and Neumann, l969; Yocum et al., 1975). Cytochrome c6 (cyt c533) was purified from *Synechocystis* (Mazor et al., 2014).

#### P700 Reduction

P700 reduction was measured in a Quartz cuvette, containing: 1 ml reaction mix (20 mM Tricine-NaOH pH 8, 5 mM MgCl_2_ and 0.05% n-Dodecyl β-maltoside), 10 μmol Ascorbate, 100 nmol Methyl viologen, 16 μg chlorophyll PSI, and 50 pmol Pc of *Synechocystis* PCC SP 6803 or 50 pmol Cyt c_6_ (Cyt C533). P700 photo-oxidation and re-reduction by Pc and Cyt c_6_ was measured by JTS-10 spectrophotometer by illuminating the sample with red light (705 nm) for 5 s. Changes in absorbance were measured by 2 ms of LED light flashes (700 nm passed through a 705 nm interference filter).

#### NADP^+^ Photo-Reduction Activity Assay

In a Quartz cuvette, 1 ml reaction mix (20 mM NaCl, 10 mM Tricine NaOH pH 8, 0.5 mM MgCl_2_) was supplemented with 20 μmol ascorbate, 125 μg Ferredoxin, 8.8 μg FNR, 1 μmol NADP^+^ (Roche Diagnostics), 14 nmol Pc, and PSI (14.4 μg chlorophyll). NADPH accumulation was measured at 340 nm (𝜖 = 6220 M^-1^ cm^-1^) by Cary 60 spectrophotometer (Agilent technologies) under continuous illumination with 660 nm LED light (600 μE). The activity was calculated as μmol NADPH/(mg chl⋅Hr).

## Results

As we previously reported, the addition of a single histidine at the C-terminus of PsaL (PsaL^HIS^) was sufficient to turn the main fraction of PSI in the mutant strain from trimers to monomers caused by an interference to the trimer formation (Malavath et al., 2018). This allowed us to examine PSI monomers structure and function in comparison to PSI trimers.

### Kinetic Measurements

**FIGURE 1:**
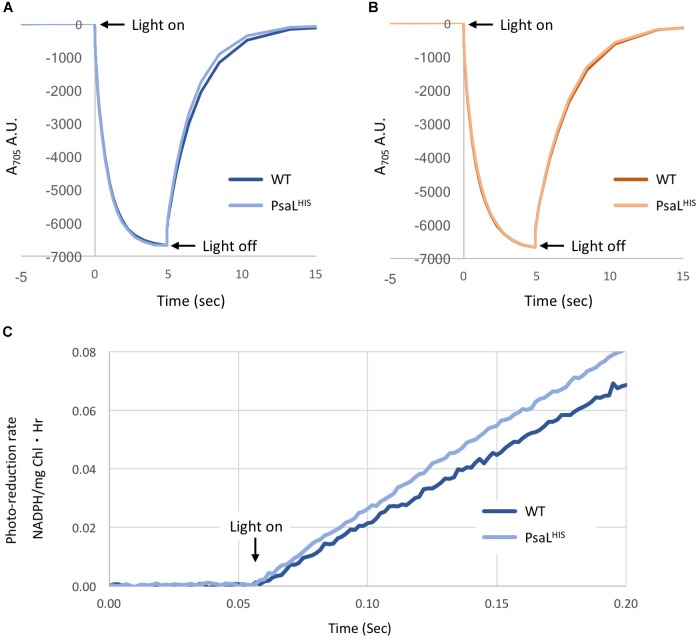
Kinetic measurements of PSI from WT and PsaL^HIS^ strains. P700 re-reduction – A reaction mix containing ascorbate and methyl viologen as electron source and terminal electron acceptor, respectively, was supplemented with 16 μg Chl PSI and 50 pmol of either Plastocyanin **(A)** or Cytochrome c_6_ **(B)**. The mix was illuminated with red light (705 nm) for 5 s and changes in absorbance were measured (*n* = 3). **(A)** Changes in A_705_ in the presence of Pc, WT – dark blue; PsaL^HIS^ – light blue. **(B)** Changes in A_705_ in the presence of Cyt c_6_, WT – dark orange; PsaL^HIS^ – light orange. **(C)** NADP^+^ photo-reduction rate – The electron transfer chain was reconstituted by mixing Pc, PSI, Fd, FNR, and NADP^+^. The mix was illuminated with 660 nm LED light (600 μE) and NADPH accumulation was measured by absorbance at 340 nm by Cary 60 spectrophotometer (*n* = 3), WT – Dark blue; PsaL^HIS^ – light blue. The calculate rate is shown as μmol NADPH/(mg chl⋅Hr).

Electron transport from PSI to ferredoxin was extensively studied (Munekage et al., 2004; Baniulis et al., 2008). Recently a crystal structure of trimeric PSI in complex with ferredoxin was published (Kubota-Kawai et al., 2018). The electron donation to oxidized P700 by reduced plastocyanin and cytochrome c_6_ was also studied using the trimeric wild-type PSI (Hippler et al., 1996). To compare the activity of WT PSI trimers and PsaL^HIS^ PSI monomers we performed different kinetic tests. We measured P700 reduction rates by Plastocyanin and Cytochrome c6. In the presence of 50 pmol Pc, the re-reduction t_1/2_ of WT and PsaL^HIS^ strains were 1.16 ± 0.0001 s and 1.04 ± 0.012 s, respectively. In the presence of 50 pmol Cyt C_6_ the re-reduction t_1/2_ were 1.33 ± 0.014 s and 1.27 ± 0.021 s for WT and PsaL^HIS^ strains, respectively. All experiments were repeated three times (*n* = 3). Our results indicate that for both Pc and Cyt c_6_, the t_1/2_ values of P700 re-reduction are very similar in WT PSI trimers and PsaL^HIS^ PSI monomers (Figures 1A,B).

Moreover, we tested NADP^+^ photo-reduction by PSI using saturated amounts of plastocyanin, ferredoxin and ferredoxin-NADP-reductase, where ascorbate served as an electron donor. There was no significant difference in NADP^+^ reduction rates between WT and PsaL^HIS^ (323.24 ± 48.37 μmol NADPH/(mg chl⋅Hr) and 374.1 ± 30.21 μmol NADPH/(mg chl⋅Hr), respectively) (Figure 1C).

### Achieving an Intact PSI Monomer Model

**FIGURE 2:**
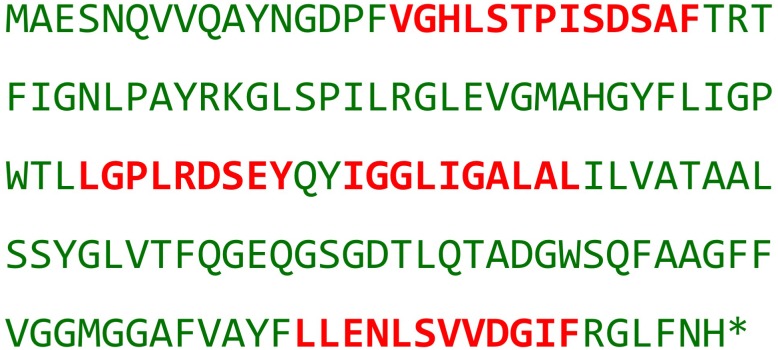
PsaL^HIS^ identification by Mass-spectrometry. Presented is the amino acid sequence of subunit PsaL^HIS^; red – fragments of PsaL^HIS^ identified by MS analysis.

The previous attempt to produce a crystal structure of PSI monomer from *Synchocystis* resulted in a PSI model lacking PsaI and PsaL, and therefore deficient of the trimerisation site (Mazor et al., 2014). In PsaL^HIS^ a single histidine addition was presumed to cause a minimal disturbance to the complex’s composition, thus allowing PSI monomer purification. Indeed, in the new PsaL^HIS^ strain the PSI trimer fraction has decreased considerably and the presence of PsaL in the complex monomers was verified by MS (Figure 2).

After screening hundreds of PsaL^HIS^ PSI crystals, we collected data sets from a few dozen crystals which diffracted to low resolution. When examined, none of the data sets could be used individually to produce an adequate model due to poor signal to noise ratio, variations in unit cell values and high anisotropicity.

Using AIMLESS we were able to combine five individual datasets to ultimately create a 4 Å dataset (Winn et al., 2011). Anisotropicity was still a major problem after combining the sets, so it was corrected in the unified dataset by the STARANISO server (Tickle et al., 2016).^1^ These crystals had a P212121 symmetry and have all displayed a similar unit cell dimensions of a 124.32, b 178.66, c 181.45, and α, β, γ 90, 90, 90 on average. Table 1 shows the x-ray collection and refinement statistics, prior to the anisotropic correction applied on the dataset. The map and model are available in the PDB under the ID 6HQB.

**Table 1:** Statistics for the combined dataset of *Synechocystis* PSI PsaL^HIS^ monomer.

Data collection	
Beamline	SLS X06SA PXI, ESRF ID23-2, BESSY II MX-14.1
Wavelength (Å)	0.999, 0.873. 0.918
Resolution (Å)	49.5–4
Space group	P212121

**Unit cell dimensions**	

a, b, c (Å)	124.32, 178.66, 181.45
α, β, γ	90, 90, 90
Measured reflections	839570 (97420)
Unique reflections	34852 (4494)
Rpim (%)	0.092 (0.437)
< I/σ(I) >	4.9 (1.9)
CC1/2	0.983 (0.759)
Completeness (%)	99.7 (98.5)
Redundancy	24 (21)

**Refinement statistics**	

Rwork/Rfree	0.255/0.295
No. of chains	11
No. of ligands	130
Average B-factor (Å^2^)	126.2

**R.M.S deviations**	

Bond angles	1.3
Bond lengths	0.004

**Ramachandran statistics**	

Favored region (%)	91.45
Allowed region (%)	7.6
Outliers region (%)	0.95
Clashscore	14.6


### Crystal Structure of PsaL^HIS^ Monomer

**FIGURE 3:**
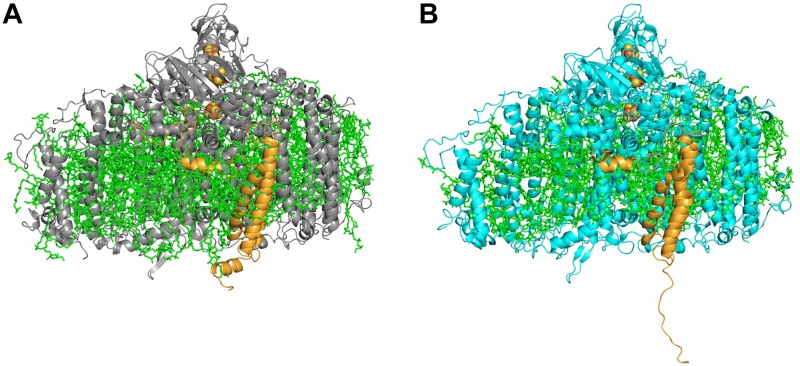
Side view of PSI monomer. **(A)** A representative monomer derived from the WT PSI trimer. **(B)** PSI PsaL^HIS^ monomer. In the mutated PSI **(B)** the C-terminus of PsaL (orange) is untethered and lost its characteristic short helix form, found in the WT PsaL **(A)**.

Cyanobacterial PSI is usually found in two oligomeric states – trimeric and monomeric, though a tetrameric form has been demonstrated (Watanabe et al., 2011; Li et al., 2014; Semchonok et al., 2016). In the WT strain of *Synechocystis* PSI is mainly found in the trimeric form. We demonstrated that upon addition of a single histidine at the C-terimnus of PsaL, the dominant form of PSI super-complex was altered from a trimer to a monomer (Figure 3) (Malavath et al., 2018).

**FIGURE 4:**
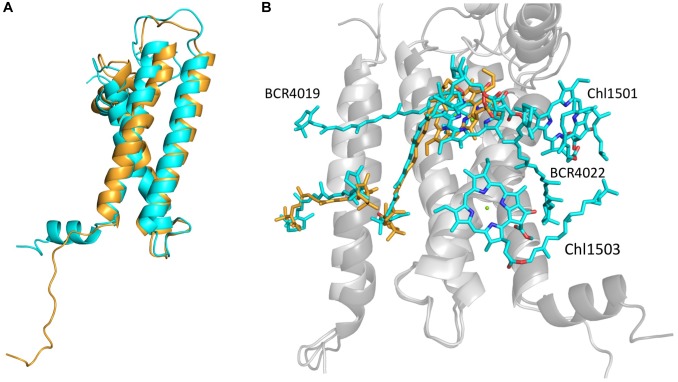
**(A)** Backbones of PsaL^HIS^ (orange) and trimer PsaL (cyan) alignment. **(B)** PsaL, PsaI and their ligands from PsaL^HIS^ (backbone – light gray; ligands – orange) are aligned to their correspondents from trimeric PSI (backbone – dark gray; ligands – cyan). The PsaL^HIS^ model is missing 2 Chlorophyll a molecules and 2 β-carotenes present in the trimer model.

As could be expected, the most drastic changes occurred in the structure and associated ligands of PsaL. While the wild-type PsaL coordinates three chlorophyll a molecules (L1501, L1502 and L1503) and two β-carotenes (L4019 and L4022), only chlorophyll L1502 was modeled into the electron density map of PsaL^HIS^ monomer. β-carotene L4022 is located outside the PSI cylinder and in the trimeric structure it is wedged between chlorophylls L1501 and L1503 and two neighboring PsaL subunits, possibly binding them together. PsaL’s second β-carotene, L4019, is encompassed on all sides by chlorophylls L1502 and B1239 on one end and PsaL and PsaI on the outer edge. Both chlorophyll a molecules L1501 and L1503, located at the outskirts of PsaL and coordinated by its first helix. It is reasonable to assume that any shift in the location of PsaL, which was observed in our monomeric structure, could interfere with ligand binding or prevent it entirely (Figure 4B).

The C-terminus region of the PsaL in the native trimeric PSI (ASN144-ASN157) creates a short helix which can interact with the neighbor monomer’s PsaL and assist in trimerisation stabilization, however, the addition of a terminal histidine has disrupted the short helical structure and created a loose C-terminus in PsaL (Figure 4A).

The largest dislocation and structural differences in the PSI subunits was observed in PsaM, PsaI and PsaL which are all located at the trimerisation area. Despite a shift of 2–3 Å in PsaI location toward PsaM and away from PsaL, both carotenoids that were coordinated by this subunit remained in their position and orientation. However, a dislocation of about 1 Å in PsaM from its original position caused carotenoid M4021 to interact exclusively with PsaM whereas in the native form it is coordinated by both PsaM and PsaI.

## Discussion

PSI has been extensively studied for many years in hope of elucidating the way by which it converts sunlight to chemical energy (Grotjohann and Fromme, 2005; Qin et al., 2015). Previous studies demonstrated that inactivation of PsaI or PsaL genes in S. 6803 resulted in the absence of PsaL from the PSI complex in S. 6803. These studies showed the importance of PsaI in PsaL’s integration into the complex and PsaL’s role in PSI trimerisation (Chitnis et al., 1993; Xu et al., 1995).

The goal of this project was to determine which differences if any, exist in the structure or function between PSI monomers and trimers. To that end we created a new *Synechocystis* S.6803 strain, PsaL^HIS^, in which the monomer is the larger fraction.

Structurally, several changes were observed at the trimerisation region of the complex. In the WT PsaL, the C-terminus is fixed in a compact α-helix form, however, in PsaL^HIS^ it is untethered. The addition of a single histidine at the end of the sequence is not expected to interrupt the formation of the protein secondary structure. This emphasizes the importance of PsaL’s C-terminus in PSI trimerisation, as it has to maintain the integrity of its secondary structure in order for trimerisation to occur. Moreover, the WT PsaL contains three chlorophyll a molecules and two β-carotenes, whereas the PsaL^HIS^ monomer contains only one chlorophyll a molecule and no β-carotene molecules. It is possible that the presence of these ligands contribute to the structural stability that holds the C-terminus in its place. An alternative hypothesis is that the lack of stable secondary or tertiary structures prevents the integration of the ligands into the protein. Whichever the case may be, the results suggest that these ligands, that are possibly incorporated during trimerisation, might play a structural role and not just participate in excitation energy transfer dissipation.

Despite the change to PSI super complex’s oligomeric state in the new PsaL^HIS^ strain, the kinetic tests we preformed showed no significant effect on the activity of PSI. This finding coincides with the structure obtained from the mutant crystals, which shows no critical changes in the complex with the exception of the trimerisation site.

In the recently published WT PSI model, we reported that PsaL also hold a putative Ca^2+^ ion and several phospholipids (Malavath et al., 2018). We suggested that this Ca^2+^ ion contributes to the trimer formation and stabilization by holding two neighboring PsaL subunits together.

Our limited resolution does not allow to ascertain that the ligands mentioned had not been incorporated to the structure, or that they had diffused out of the structure since they were located at the outskirts of the super-complex. Although our low resolution structure does not permit us to identify the exact ligand composition of the PsaL^HIS^ monomer, we can assume there is a specific set of ligands that defines the main monomer. This putative monomer is used to assemble either the trimeric PSI or modified to create other asymmetric monomers for which our model can be used as a representative.

## Data Availability Statement

The following dataset was generated:

**Table 2:** 

**Author(s)**	**Year**	**Dataset title**	**Dataset ID**
Netzer-El S.Y.	2018	Monomeric	PDB ID 6HQB
Caspy I		cyanobacterial	
Nelson N		photosystem I	

## Author Contributions

SN-E performed the biochemical procedures, isolation of PSI, crystallization, data collection, kinetic measurements, discussion, and writing the paper. IC performed the structure determination, data collection, discussion, and writing the paper. NN supervised the study, collected the data, and participated in the experiments.

## Conflict of Interest Statement

The authors declare that the research was conducted in the absence of any commercial or financial relationships that could be construed as a potential conflict of interest.

## Footnotes

1 http://staraniso.globalphasing.org/cgi-bin/staraniso.cgi
